# Time Trends for Incidence and Net Survival of Cervical Cancer in Sweden 1960–2014—A Nationwide Population-Based Study

**DOI:** 10.1158/1055-9965.EPI-21-1323

**Published:** 2022-06-02

**Authors:** Avalon Sundqvist, Louise Moberg, Paul W. Dickman, Thomas Högberg, Christer Borgfeldt

**Affiliations:** 1 Department of Obstetrics and Gynecology, Skåne University Hospital Lund, Lund University, Sweden.; 2 Department of Medical Epidemiology and Biostatistics, Karolinska Institute, Stockholm, Sweden.; 3 Division of Oncology, Department of Clinical Sciences Lund, Lund University, Sweden.

## Abstract

**Background:**

The aim was to investigate time trends for incidence and long-term net survival in the morphologic subtypes and stages of cervical cancer in Sweden during the period 1960 to 2014.

**Methods:**

Women with invasive cervical cancer were identified through the Swedish Cancer Registry. Incidence and net survival were calculated according to morphology, age at diagnosis, and FIGO stage at diagnosis.

**Results:**

In total, 29,579 cases of invasive cervical cancer between 1960 and 2014 were included. The age-standardized incidence for squamous cell carcinoma (SCC) decreased until 2000; thereafter, the incidence rate stagnated, and a small increase was found in 2014. The incidence of adenocarcinoma continuously increased. The age-standardized 5-year net survival increased. However, decreasing net survival with increasing age was found. A higher stage at diagnosis showed a worse net survival. SCC and adenocarcinoma did not statistically differ as regards net survival in the last years of the study.

**Conclusions:**

Age-standardized 5-year net survival improved between 1960 and 2014. A positive trend for short- and long-term net survival was seen for women ages 18 to 64 years but long-term net survival for women ≥75 years decreased. In this study, age and FIGO stage at diagnosis were found to be important prognostic factors in determining net survival. The morphologies, SCC, and adenocarcinoma did not statistically differ as regards net survival in the last years of the study.

**Impact:**

This study demonstrates longitudinal data on cervical cancer in Sweden for over 50 years with sub analyses on morphology, age, and stage at diagnosis.

## Introduction

Cervical cancer is the fourth most common cancer among women worldwide and leads to a large number of cancer-related deaths every year (1). In Sweden, cervical cancer used to be the second most common cancer among women (2), but due to the introduction of a nationwide organized cervical screening program in 1966 (3) the incidence has decreased by nearly 50% and the mortality by almost 70% (1). During more than 50 years of nationwide screening, the screening program has developed from the initial use of Papanicolaou tests, to the use of liquid-based cytology (4) and since 2015, the National Guidelines recommend testing for high-risk types of human papillomavirus (hr-HPV; ref. 3). Furthermore, in 2012 vaccinations against hr-HPV were introduced as part of the Swedish childhood vaccination program for all girls, and in 2020 boys were included as well (3, 5). In summary, preventive work for cervical cancer is well established in Sweden, but despite this, during the last decade the decrease in incidence of cervical cancer has stagnated (6, 7).

Cervical cancer can be divided into squamous cell carcinoma (SCC), adenocarcinoma, and other rarer subtypes. SCC is the most common subtype (1), but the incidence of adenocarcinoma is increasing (8, 9). Known co-factors that are associated with risk of cervical cancer and acquisition and persistence of hr-HPV infections are early debut of first intercourse, multiple sexual partners, other sexually transmitted diseases, young age at first birth, high parity, long-term use of oral contraceptives, and smoking (10, 11). With increasing incidence of adenocarcinoma it is of interest to discover if the survival outcome will be affected. However, there are conflicting results on whether or not the tumor histology is a prognostic factor for survival outcome (12–14).

The 5-year relative survival (RS) for cervical cancer has increased in Europe, the Nordic countries and the United States during the last decades, although not for stage IV disease in the United States 1983 to 2009 (7, 13, 15–17). Multiple factors are suggested to be related to the survival rates of cervical cancer, including residential factors such as access to organized screening and treatment as well as individual factors comprising year, stage and age at diagnosis, and histopathologic subtype (13, 18). Previous studies have proposed that clinical stage is one of the most important independent prognostic factors (13, 18). Some studies state that age at diagnosis is a prognostic factor (7, 13, 19) as well as if the cancer was discovered in screening or if the woman had symptoms (20, 21). However, there are few studies investigating the individual factors in relation to net survival over a prolonged period.

The aim of this study was to investigate time trends for incidence and long-term net survival in the morphologic subtypes and FIGO stages of cervical cancer in Sweden during the period from 1960 to 2014 using data from the Swedish Cancer Registry (SCR).

## Materials and Methods

### The SCR

The population-based nationwide SCR started registration in 1958. The register contains data about gender, personal identification number, domicile, date of diagnosis, tumor site, histologic type, FIGO stage at diagnosis (since 2004), basis of diagnosis, reporting hospital, and department. The SCR receives data once yearly from six regional registries associated with regional cancer centers covering the whole country. It is compulsory for clinicians, pathologists, and cytologists to independently report all patients with premalignant and malignant conditions and certain benign tumors. This ensures the high coverage of the registry (22). In 1958 to 1986 the tumor site was coded in International Classification of Diseases-7 (ICD-7), in 1987 to 1992 in ICD-9, in 1993 to 2004 in International Classification of Disease of Oncology 2 (ICD-O/2) and from 2005 in ICD-O/3. The codes are translated to ICD-7 for the whole period to enable comparability over time. Morphological codes are available during the whole period as the older World Health Organization (WHO) histology code (WHO/HS/CAN/C24.1). A more detailed morphology coding according to ICD-O/2 was used from 1993 to 2004, and since 2005, ICD-O/3 has been used. All gynecological tumors are staged according to the International Federation of Gynecology and Obstetrics (FIGO; ref. 22). Since 2004, information on FIGO stage has been collected. The completeness of the SCR is over 95% (23) and 98% of cancer cases are verified by morphology (24). Because Sweden has a system in which all residents have a unique identification number, the follow-up of the patients in the registry is close to complete up to the time of death or emigration (25).

### Cohort

**Figure 1.**
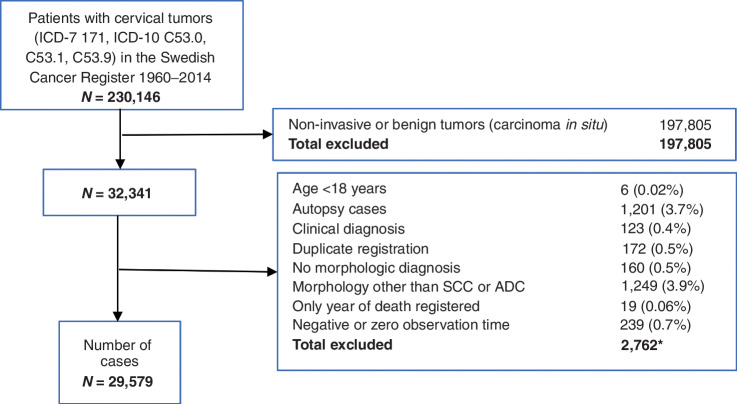
Flow chart. ICD-7 171 tumor of cervix uteri. ICD-10 C53.0 malignant tumor of the endocervix, C53.1 malignant tumor of the exocervix, C53.9 unspecified localization of malignant tumor of cervix uteri. *Some cases fulfilled more than one exclusion criteria.

All women with a cervical tumor (ICD-7 171, ICD-10 C53.0, C 53.1, and C53.9) registered in the period 1960 to 2014 were identified and included in the initial cohort. Non-invasive or benign tumors (carcinoma in situ) were excluded from the analysis cohort. The women were then matched with the Swedish Death Registry up until May 7, 2020 (Fig. 1). We did not exclude any women based on previous or subsequent diagnoses of other tumors because this could have introduced a bias (26, 27). For the analyses the women were grouped according to morphology; SCC and adenocarcinoma, age at diagnosis; 18 to 44, 45 to 54, 55 to 64, 65 to 74, and 75+ years and FIGO stage; I–II and III–IV.

### Statistical analyses

To facilitate international comparisons, incidence rates were age-standardized to the World Standard population 2011 (28). Survival time was measured from date of diagnosis until date of death, date of emigration, or May 7, 2020. We estimated net survival in an RS framework, which is a standard approach for population-based studies of cancer patient survival (29). Net survival is the probability of surviving beyond a given time without dying of the disease under study in a hypothetical scenario where the disease under study is the only possible cause of death. Net survival is preferred for comparing cancer patient survival between countries or within a single country because it is independent of mortality due to other causes. There are two frameworks available to estimate net survival; relative- and cause-specific. RS is generally favored for population-based studies (30).

We estimated net survival using flexible parametric models (31). Expected mortality rates for women, stratified by age and calendar year, were retrieved from the Human Mortality Database (http://www.mortality.org) based on data from Statistics Sweden. The model included the main effects of age at diagnosis (categorized as 18–44, 45–54, 55–64, 65–74, and ≥75 years), morphology (SCC and adenocarcinoma), year of diagnosis (1960–2014) as restricted cubic spline with three degrees of freedom, all three two-way interactions between age, year of diagnosis, and morphology, and time-varying effects of age, morphology, age*morphology and age*year. The baseline cumulative excess hazard was modeled as a restricted cubic spline with five degrees of freedom and the time-varying effects were modeled using two degrees of freedom. On the basis of this model, we estimated temporal trends in net survival within each age group along with age-standardized net survival using the International Cancer Survival Standard population number 2 (32). We also estimated the difference in age-standardized net survival between the two morphologic types. The analytic process for age-standardization is illustrated (using publicly available data for melanoma where the exposure of interest is sex rather than morphology) at http://pauldickman.com/software/stata/age-standardise-standsurv/. The statistical analyses were performed using Stata version 16 (StataCorp.).

### Data availability

The individual-level data used in this study are maintained by the SCR and are classified as sensitive personal data according to the General Data Protection Regulation (GDPR). Access to the data can be obtained according to the exception in GDPR that allows processing of sensitive personal data for research provided that an ethical permit exists. Raw data are available by applying to the Swedish National Board of Health and Welfare.

### Ethical approval

This study was approved by the Regional Ethical Review Board, Lund (DNR 2015/789). This study was a retrospective register study, and no patient consent was required. The benefit of the study was considered to be greater than the potential harm to the patients. This study was performed in accordance with the Declaration of Helsinki.

## Results

In total, 230,146 patients with cervical tumors (ICD-7 171, ICD-10 C53.0, C53.1, and C53.9) were found in the Swedish Cancer Register between 1960 and 2014. After applying the exclusion criterion listed in Fig. 1, 29,579 cases of invasive cervical cancer were included in the analysis (Fig. 1).

### Demography

The age group of 18 to 44 years was the most common age at diagnosis for the whole period and became even more common during the period of 2010 to 2014 (1960–1969 37.0% vs. 2010–2014 46.2%, *P* < 0.001). The age group of 45 to 54 years was the second most common age at diagnosis between 1960 and 1969 (29.2%), and this was still the case in 2010 to 2014 but diagnosis in the oldest age group (75+) increased during later time periods and was equally common as diagnosis at 45 to 54 years in 2010 to 2014 (14.0% vs. 16.3%, *P* = 0.04). The median age at diagnosis for 2010 to 2014 was the lowest during the observed period (range, 46–54). SCC was the most common subtype during the whole period, although the proportion and numbers of cases continuously decreased (94.0% in 1960–1969 vs. 76.9% 2010–2014, *P* < 0.001) whereas the proportion of adenocarcinoma increased almost 4-fold after 1960 (6.0% vs. 23.1%, *P* < 0.001). The proportion of missing FIGO stages decreased from 31.8% in 2005 to 2009 to 15.9% in 2010 to 2014 (*P* < 0.001; see Table 1).

**Table 1.** Demography of patient cohort.

	1960–69	1970–79	1980–89	1990–99	2000–09	2010–2014	Total
Age at diagnosis	Median years (range)	Median years (range)	Median years (range)	Median years (range)	Median years (range)	Median years (range)	Median years (range)
	48 (18–90)	54 (19–95)	54 (18–95)	51 (20–98)	51 (21–98)	46 (18–97)	51 (18–98)
	**1960–69**	**1970–79**	**1980–89**	**1990–99**	**2000–09**	**2010–2014**	**Total**
Age at diagnosis	*n* (%)	*n* (%)	*n* (%)	*n* (%)	*n* (%)	*n* (%)	*n* (%)
18–44	2,945 (37.0)	1,800 (30.0)	1,815 (36.9)	1,601 (36.4)	1,632 (39.4)	1,009 (46.2)	10,802 (36.5)
45–54	2,325 (29.2)	1,277 (21.3)	651 (13.2)	802 (18.2)	677 (16.4)	356 (16.3)	6,088 (20.6)
55–64	1,547 (19.5)	1,420 (23.7)	788 (16.0)	543 (12.3)	617 (14.9)	270 (12.4)	5,185 (17.5)
65–74	803 (10.1)	1,005 (16.8)	975 (19.8)	748 (17.0)	486 (11.7)	242 (11.1)	4,259 (14.4)
75+	329 (4.1)	489 (8.2)	687 (14.0)	707 (16.1)	727 (17.6)	306 (14.0)	3,245 (11.0)
Total	7,949 (100)	5,991 (100)	4,916 (100)	4,401 (100)	4,139 (100)	2,183 (100)	29,579 (100)
	**1960–69**	**1970–79**	**1980–89**	**1990–99**	**2000–09**	**2010–2014**	**Total**
Morphology	*n* (%)	*n* (%)	*n* (%)	*n* (%)	*n* (%)	*n* (%)	*n* (%)
Squamous cell carcinoma	7,474 (94.0)	5,466 (91.2)	4,107 (83.5)	3,504 (79.6)	3,156 (76.3)	1,678 (76.9)	25,385 (85.8)
Adenocarcinoma	475 (6.0)	525 (8.8)	809 (16.5)	897 (20.4)	983 (23.7)	505 (23.1)	4,194 (14.2)
Total	7,949 (100)	5,991 (100)	4,916 (100)	4,401 (100)	4,139 (100)	2,183 (100)	29,579 (100)
					**2005–2009**	**2010–2014**	**Total**
FIGO stage					*n* (valid %; total %)	*n* (valid %; total %)	*n* (valid %; total %)
I					902 (64.0–43.6)	1,189 (64.8–54.5)	2,091 (64.4–49.2)
II					258 (18.3–12.5)	331 (18.0–15.2)	589 (18.2–13.9)
III					161 (11.4–7.8)	189 (10.3–8.7)	350 (10.8–8.2)
IV					89 (6.3–4.3)	126 (6.9–5.8)	215 (6.6–5.1)
Missing					657 (0.0–31.8)	348 (0.0–15.9)	1,005 (0.0–23.6)
Total valid cases					1,410	1,835	3,245
Total incl. missing cases					2,067	2,183	4,250

### Age-standardized incidence

**Figure 2.**
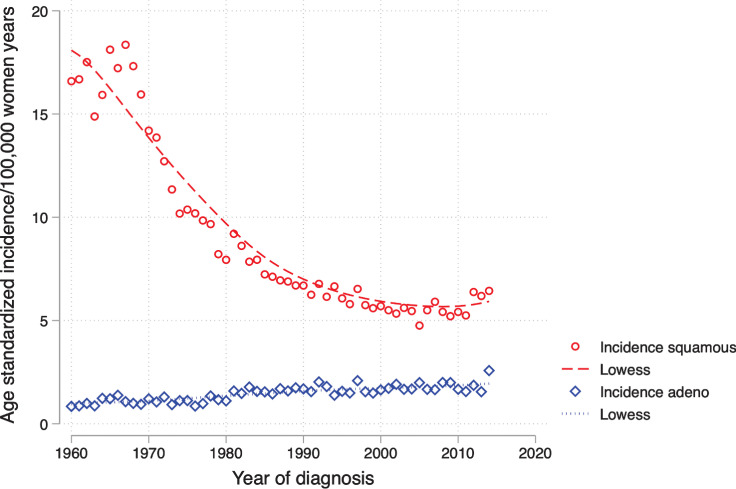
Age-standardized incidence depending on morphology. Age-standardized incidence depending on the morphology squamous cell carcinoma and adenocarcinoma per 100,000 women years standardized to the world population.

The cervical cancer incidence of SCC decreased in Sweden from 1960 to 2000; thereafter, the incidence rate stagnated but a small increase was found in 2014. The incidence of adenocarcinoma continuously increased during the whole study period; see Fig. 2.

### Age-standardized 5-year net survival

**Figure 3.**
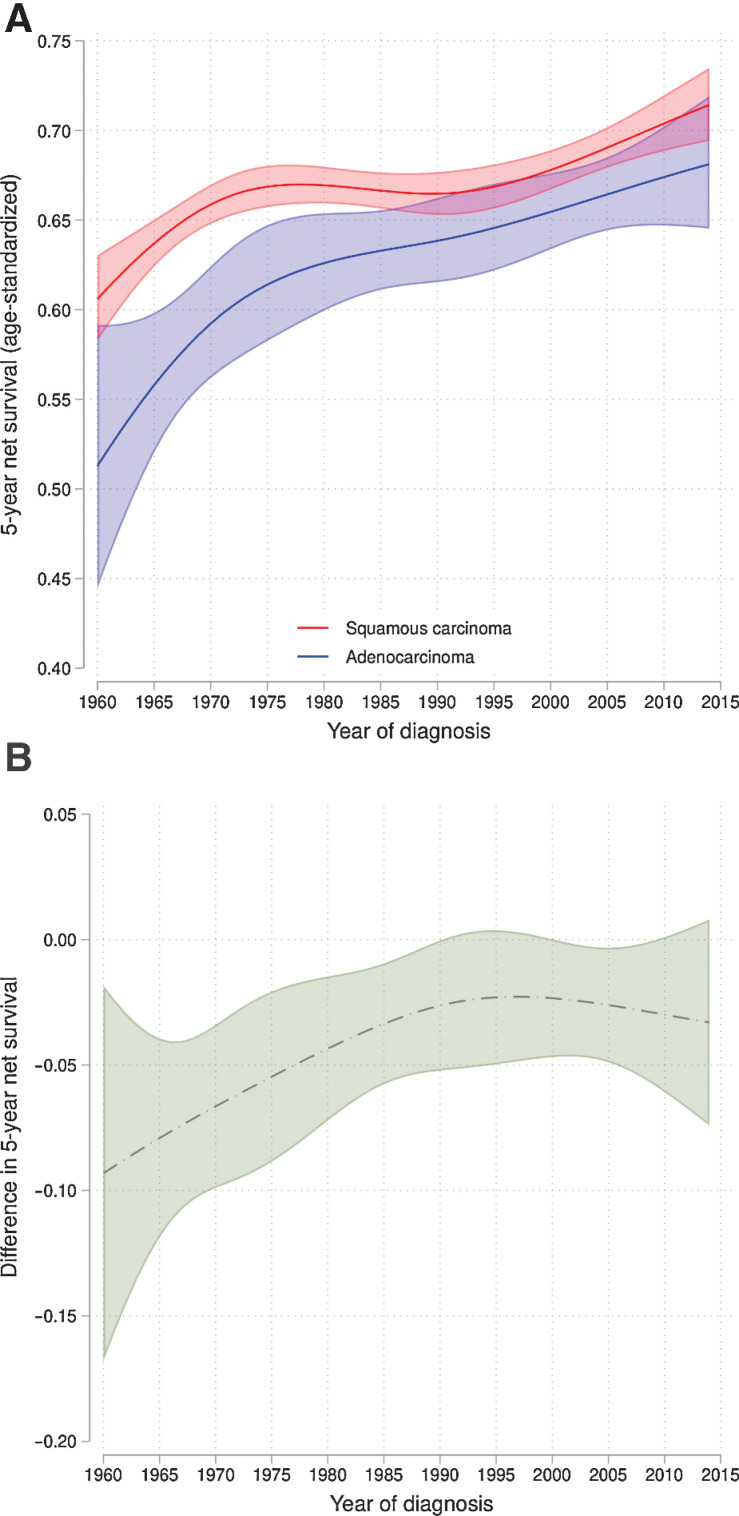
Age-standardized 5-year net survival according to morphology. **A,** age-standardized 5-year net survival for squamous cell carcinoma and adenocarcinoma with 95% confidence intervals. **B,** Difference in age-standardized 5-year net survival of adenocarcinoma and squamous cell carcinoma with 95% confidence intervals. No statistical difference in net survival was found between squamous cell carcinoma and adenocarcinoma 1990–2000 and after 2010.

The age-standardized 5-year net survival increased from 1960 to 2014 for SCC and adenocarcinoma. The greatest improvement was found for adenocarcinoma. The age-standardized 5-year net survival was somewhat higher for SCC compared with adenocarcinoma during the whole period, but the difference was very small. In the periods 1990 to 2000 and 2010 to 2014, the difference was even smaller and not statistically significant; see Fig. 3.

### Age-specific net survival

**Figure 4.**
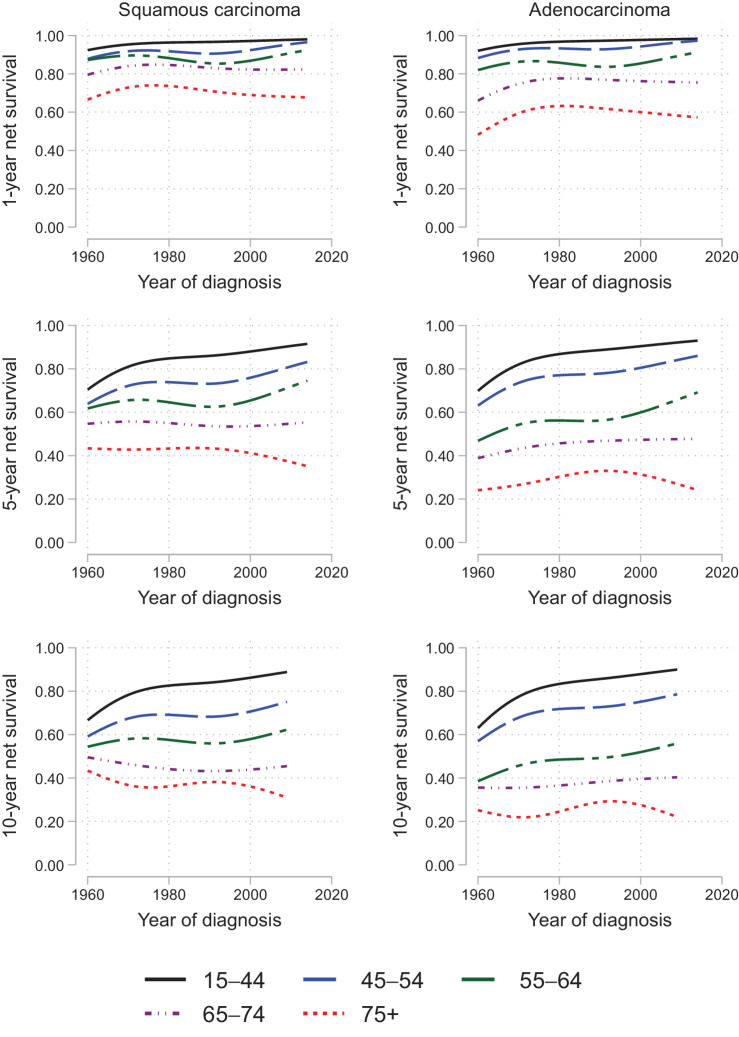
Age-specific net survival. Time trends for 1-, 5-, and 10-year net survival according to age groups and for squamous cell carcinoma and adenocarcinoma.

During the study period, decreasing long- and short-term net survival with increasing age was found for SCC and adenocarcinoma. During the period of 1960 to 2014, the 1-, 5- and 10-year net survival improved for women ages 18 to 64 years diagnosed with SCC. In older age groups the improvements were smaller and for ages ≥75 no improvement but a decrease was found for 5- and 10-year net survival since 1960. For women diagnosed with adenocarcinoma, an increase in 1-, 5- and 10-year net survival was found since 1960 in all age groups except for 5- and 10-year net survival for women ≥75 years; see Fig. 4.

### Age-standardized net survival according to morphology per stage

**Figure 5.**
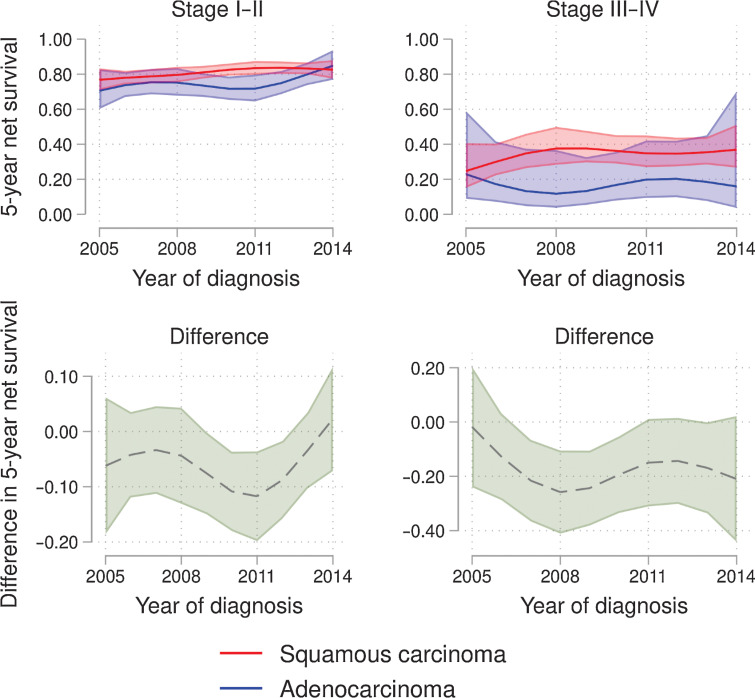
Age-standardized 5-year net survival according to morphology per stage. Age-standardized 5-year net survival according to squamous cell carcinoma and adenocarcinoma, FIGO stages I–II and III–IV during the time 2005–2014 with 95% confidence intervals (top figure). Difference in age-standardized 5-year net survival of adenocarcinoma and squamous cell carcinoma, stages I–II and III–IV during the period 2005–2014 with 95% confidence intervals (bottom figure). No statistical difference in net survival was found between squamous cell carcinoma and adenocarcinoma in stages I–II after year 2012 and neither in stages III–IV after year 2011.

Higher stage at diagnosis demonstrated a worse net survival. The 5-year net survival for stages I–II was maintained for SCC and increased for adenocarcinoma between 2005 and 2014. In 2014 both morphologies had a 5-year net survival of ≥80% and there was no statistically significant difference in net survival between the morphologies in 2005 to 2009 and 2012 to 2014. For stages III–IV the 5-year net survival increased for SCC and stayed rather stable for adenocarcinoma, with SCC demonstrating a 5-year net survival of ≥35% and adenocarcinoma of <20% in 2014. In 2005, there was no detected difference in 5-year net survival between the morphologies in stages III–IV, from 2006 to 2014, SCC demonstrated a better 5-year net survival than adenocarcinoma although from 2011 the difference was not statistically significant; see Fig. 5.

## Discussion

### Incidence trends

During the studied period, there has been a declining incidence of cervical cancer in Sweden, which has also been found in other Nordic countries, the US, Canada, Australia, New Zealand, Japan, and in several countries in Europe (7, 8, 16, 33–35). The decrease in cervical cancer incidence is due solely to declining incidence of SCC. Reasons for this decline in incidence of SCC may be the beneficial effects of an effective nationwide cervical screening program in combination with lifestyle changes (35). Several co-factors associated with risk of cervical cancer in the presence of hr-HPV have changed toward less risk for cervical cancer in Sweden. Mean parity has declined (36), age at first birth has increased (37), everyday smokers have declined (38, 39) and the use of oral contraceptives has declined (40). However, during recent years the incidence rate has stagnated and a small increase in the incidence of SCC was found in 2014. An increase in cervical cancer incidence has also been seen in Finland, Norway, the Netherlands, and among women ≤49 years of age in the UK (41–44). It has been noticed with concern by other Swedish researchers that the incidence of cervical cancer has increased (45), despite the fact that several quality indicators for cervical cancer prevention in Sweden have changed in a positive direction (46, 47). The most significant increase (30%) in cervical cancer was reported in women who had a normal cell sample in the previous screening (48). In a Swedish study, it was reported that the increase in cervical cancer incidence was seen solely in women who had attended screening and only in some regions of Sweden, suggesting regional issues with quality assurance and insufficient detection of precancerous lesions as the most possible cause of the increased cancer incidence (49). A large re-examination of previous normal cytological samples from women diagnosed with cervical cancer demonstrated that the proportion of samples that have been reclassified from normal to abnormal during re-examination has increased starting with cervical tests taken in 2009 (49, 50). In Sweden, there are some co-factors associated with risk of cervical cancer that have changed toward an increased risk. Age at first intercourse has decreased (51, 52), the number of sexual partners has increased (51), and infection with chlamydia trachomatis had an incidence peak in 2007 (53). However, because the increase in cervical cancer has not been seen among screening non-attendees and not in all regions, it is unlikely that changes in risk factors contribute to the increased cervical cancer incidence in Sweden (49).

For adenocarcinoma, the incidence has continuously increased during the study period. This trend has also been found in studies from the US, Europe, Norway, and Iceland (8, 9, 42, 54). Cytology is known to be less sensitive for detection of adenocarcinoma, which could be one explanation for the lack of effect on adenocarcinoma (55). However, a nationwide audit showed that the screening program in Sweden was effective in reducing the incidence of adenocarcinoma (20). Some state that the continuous increase in adenocarcinoma might be due to improved morphological specificity with a decrease in unspecified malignant tumors and a concurrent increase in adenocarcinoma (14). Persistent infection with hr-HPV types is the causative factor for both SCC and adenocarcinoma, although SCC is linked more to infection with HPV-16 whereas adenocarcinoma is more often associated with HPV-18 (56). HPV-16, 18, and 31 are the most common hr-HPV types in Sweden. HPV-16 has the highest prevalence, but we do not know if the prevalence of HPV-18 might have changed during the last decades (56). It would be of interest to study long-term data on HPV prevalence in Sweden, especially because the incidence of some other HPV-related cancers is increasing (penile, anal, and oropharyngeal cancers; ref. 6). Among women ages 15 to 23 years old in Stockholm, Sweden, in the period 2017 to 2018 in comparison with the period 2008 to 2010, the prevalence of both HPV 16 and 18 decreased, whereas HPV 39, 51, 52, 56 and 59 increased, irrespective of HPV vaccination status, which could be explained by herd immunity (57). A change in risk factors should also be considered, but SCC and adenocarcinoma share all risk factors except smoking, which has only been associated with increased risk for SCC (10); therefore, a change in risk factors should not affect adenocarcinoma solely. We cannot state the exact reason for the increased incidence of adenocarcinoma but hopefully, in coming years the incidence of adenocarcinoma will begin to decrease because of HPV vaccination and HPV-based primary screening, which provides better protection against invasive cervical cancer, including adenocarcinoma compared with cytology-based screening (58).

### Wider age span—median age

During the studied period there was a significant increase in cervical cancer diagnosis among women 18 to 44 years and women 75+ years. In late 1960s, the recommendation was to screen women ages 30 to 49 years old (2). In 1998, the national recommendations of the upper screening age changed to 60 years (59) and in 2015 recommendations were that women ages 23 to 64 years should be screened with recall every year up to 70 years of age if no sample was registered at the age of 64 (3). Thus, it took several decades for the oldest women to become part of the screening program, which may explain why more women are being diagnosed at older ages in later time periods. Moreover, life expectancy of women in Sweden has increased from 76 years in 1961 to 1970 to 85 years in 2019 (60). Thus, the number of elderly women in Sweden has increased, which also will be reflected in more cases of cancer in this age category. More women are also being diagnosed in the youngest age category, which is mirrored by a decrease in median age. An increase in cervical cancer diagnosis among younger women has been seen in other studies, especially among women diagnosed with adenocarcinoma (8, 61). As women included in the childhood vaccination program against HPV reach screening age, a decrease in the proportion of cervical cancer in the youngest age group is expected (62).

### Improved age-standardized 5-year net survival

Between 1960 and 2014 there was an improved 5-year net survival for cervical cancer. The age-standardized 5-year net survival for SCC and adenocarcinoma in 2014 was approximately 70%, which is higher compared with the age-standardized 5-year net survival of most countries in Europe in the period 2010 to 2014 (range, 54.8%–80.1% in 28 countries in Europe 2010–2014; ref. 63). The greatest improvement of 5-year net survival of both SCC and adenocarcinoma was at the beginning of the implementation of cervical screening between 1960 and mid-1970s. Between 1980 and 2000 there was almost no improvement in 5-year net survival of SCC, and a continuously increase, though less marked in comparison with earlier periods, for the 5-year net survival of adenocarcinoma. A less marked improvement in survival during recent decades has been observed in northern and western European countries. A common factor for these countries was a high survival rate already in the 1980s (13). The most important factor regarding the improved survival is probably the cervical screening program enabling detection of early cervical cancer associated with better survival (1). However, a previous study found that 64% of all cervical cancer cases and 83% of advanced cases of cancer in Sweden were found in screening non-attendees (20). For non-attending women, an improved participation rate in the screening program to gain a stage shift, as well as improved treatment, are important factors in improving the net survival. During the 1960s, treatment for cervical cancer consisted of surgery and/or radiotherapy, which had relatively successful results for early-stage disease. However, for late-stage disease, treatment options were less developed (64). A great change in cervical cancer treatment during the period of 1960 to 2014 occurred in 1999 when four articles reported improved survival with a combination of cisplatin and radiotherapy in treatment of patients with locally advanced disease (15). After the year 2000, the curves for 5-year net survival shifted toward a greater increase in net survival, especially for SCC, which might be ascribed the effects of the treatment improvement. For women with early-stage disease the treatment options are similar to those in 1960, surgery or radiotherapy, but surgical techniques and the delivery of radiotherapy have improved (65).

### Net survival and morphology

There have been conflicting results concerning whether the tumor histology is a prognostic factor for survival outcome or not (12, 14, 66). In this study, the 5-year net survival was better for SCC than adenocarcinoma during the whole study period, but the difference was very small. During the period of 1990 to 2000 and 2010 to 2014, the difference was even smaller and not statistically significant. During the last decades and currently, there has been no difference in the national treatment guidelines for SCC and adenocarcinoma. Our data support these guidelines because the differences in net survival between the morphologies have been small in the last 30 years and the cause of the disease is thought to be similar HPV-induced tumor gene suppression carcinogenesis (1).

### Net survival and age

Several studies have found decreasing survival with increasing age, which is supported by our results (19, 67, 68). Elderly women are diagnosed in more advanced stages, which is why their prognosis is poor (21). A recent study investigated primary treatment patterns of cervical cancer in Sweden. In this study, 6% of the study population was not given any primary treatment, the median age in this group was 81 years and 63% had at least FIGO stage IIB (65). Since the 1960s 1-, 5- and 10-year net survival has improved for women ages 18 to 64 years diagnosed with SCC, but for women ≥75 years, 5- and 10-year net survival has decreased. In other words, not only do older women with SCC have a worse net survival in comparison with younger women, but also during the last 50 years the long-term net survival has decreased. For adenocarcinoma, 1-, 5- and 10-year net survival has improved for all age categories during the studied period except for 5- and 10-year net survival for women ≥75 years. To our knowledge, this study is the first to analyze time trends of net survival in different subtypes and age groups of cervical cancer. The poor net survival results in elderly women and the longer life expectancy in women indicate the need to further evaluate if screening with HPV analyses in women should be prolonged to the age of 75. However, previous studies have shown the importance of including information about the woman's previous screening history to the question of when it is safe to stop cervical screening (69, 70). Furthermore, the change to HPV-based screening does offer a better protection than cytology among older women that also must be considered (71).

### Stage

In this study, a clear relation between inferior net survival with higher FIGO stage at diagnosis was seen, which is in accordance with previous studies (13, 15, 19, 67). For early-stage cervical cancer some studies have suggested a similar survival between adenocarcinoma and SCC (66, 72), whereas others have suggested an inferior survival outcome for adenocarcinoma (12, 73, 74). For advanced-stage cervical cancer, the data are more limited. Two previous studies found a poorer survival for advanced-stage adenocarcinoma compared with SCC (12, 75). Another study found a small but statistically significant improved survival for SCC for regional disease, but no difference in survival between the morphologies for localized or distant disease (14). In our large population-based study, we found no difference between SCC and adenocarcinoma in net survival in stages I–II in 2014. In stages III–IV, SCC demonstrated a better 5-year net survival from 2006 to 2014 but from 2011 the difference was not statistically significant. During the study period, there were no major changes in treatment of any stage but the results in this study indicate the need for improved treatment in stage III and/or IV of cervical cancer, especially for adenocarcinoma.

### Strengths and limitations

This is a nationwide population-based study presenting information about cervical cancer in Sweden for over 50 years, which is a major strength of the study. Reporting to the SCR is mandatory, and the coverage is high (>95%), in combination with morphologic verification of the diagnosis in >98% of cases (2, 24). Incidence rates were age-standardized to the World Standard population that enables international comparison and minimized confounding from changing population patterns. Stage was not included in the SCR until 2004; therefore, stage-specific time trends for early time periods could not be calculated, which is a limitation. Furthermore, the absence of central pathology is a limitation of the study. Improvements in histopathological diagnosis since 1960 are an important bias to consider.

### Conclusions

The age-standardized incidence of SCC continuously decreased in Sweden between 1960 and 2000; thereafter, the incidence rate stagnated, and a small increase was found in the last year of the study period, in 2014. For adenocarcinoma, the incidence increased during the whole study period. Most women were diagnosed with cervical cancer between the ages of 18 and 44 years, but in recent years the diagnosis among women over 75 years has increased. Age-standardized 5-year net survival improved between 1960 and 2014. The short- and long-term net survival decreased with increasing age at diagnosis. A positive trend for net survival was seen for all women ages 18–64 years of age but long-term net survival for women ≥75 years was decreased, suggesting the need to further evaluate whether screening with HPV analyses in some women should be prolonged. In this study, age and FIGO stage at diagnosis were found to be important prognostic factors in determining net survival. SCC and adenocarcinoma showed no clinically significant difference regarding net survival in most of the years during the study period and did not statistically differ regarding net survival in the last years of the study.
